# Supplementary material for: Computational Protein Design with Deep Learning Neural Networks
Source: Sci Rep. 2018 Apr 20;8:6349. doi: 10.1038/s41598-018-24760-x (PMC5910428; doi:10.1038/s41598-018-24760-x)
Supplement: Supplementary file 1 — Supporting Information [file 41598_2018_24760_MOESM1_ESM.docx]

**Supporting Information**

**Computational Protein Design with Deep Learning Neural Networks**

Jingxue Wang^1^, Huali Cao^1^, John Z.H. Zhang^1-4^, and Yifei Qi^1,2*^

*^1^Shanghai Engineering Research Center of Molecular Therapeutics and New Drug Development, School of Chemistry and Molecular Engineering, East China Normal University, Shanghai, 200062, China*

*^2^NYU-ECNU Center for Computational Chemistry at NYU Shanghai, Shanghai 200062, China*

*^3^Department of Chemistry, New York University, NY, NY 10003, USA*

*^4^Collaborative Innovation Center of Extreme Optics, Shanxi University, Taiyuan, Shanxi 030006*

Correspondence

Yifei Qi: [yfqi@chem.ecnu.edu.cn](mailto:yfqi@chem.ecnu.edu.cn)

Table S1. Number of data samples (clusters) in the three data sets with different number of neighboring residues (*N*) from 10 to 30.

| Identity cutoff | *N*=10 | *N*=15 | *N*=20 | *N*=25 | *N*=30 |
| --- | --- | --- | --- | --- | --- |
| 30% | 2,063,289 | 1,944,301 | 1,844,462 | 1,759,860 | 1,687,397 |
| 50% | 2,996,686 | 2,837,472 | 2,702,161 | 2,587,105 | 2,488,142 |
| 90% | 3,797,494 | 3,603,588 | 3,438,332 | 3,297,570 | 3,175,375 |

Table S2. PDB ID and chain ID of the 50 test proteins in SPIN.

| 1eteA, 1v7mV, 1y1lA, 3pivA, 1or4A, 2i39A, 4gcnA, 1bvyF, 3on9A, 3vjzA, 3nbkA, 3l4rA, 3gwiA, 4dkcA, 3so6A, 3lqcA, 3gknA, 3nngA, 2j49A, 3fhkA, 2va0A, 3hklA, 2xr6A, 3ii2A, 2cayA, 3t5gB, 3ieyB, 3aqgA, 3q4oA, 2qdlA, 3ejfA, 3gfsA, 1ahsA, 2fvvA, 2a2lA, 3nzmA, 3e8mA, 3k7pA, 3ny7A, 2gu3A, 1pdoA, 1h4aX, 1dx5I, 1i8nA, 2cviA, 3a4rA, 1lpbA, 1mr1C, 2xcjA, and 2xdgA. |
| --- |


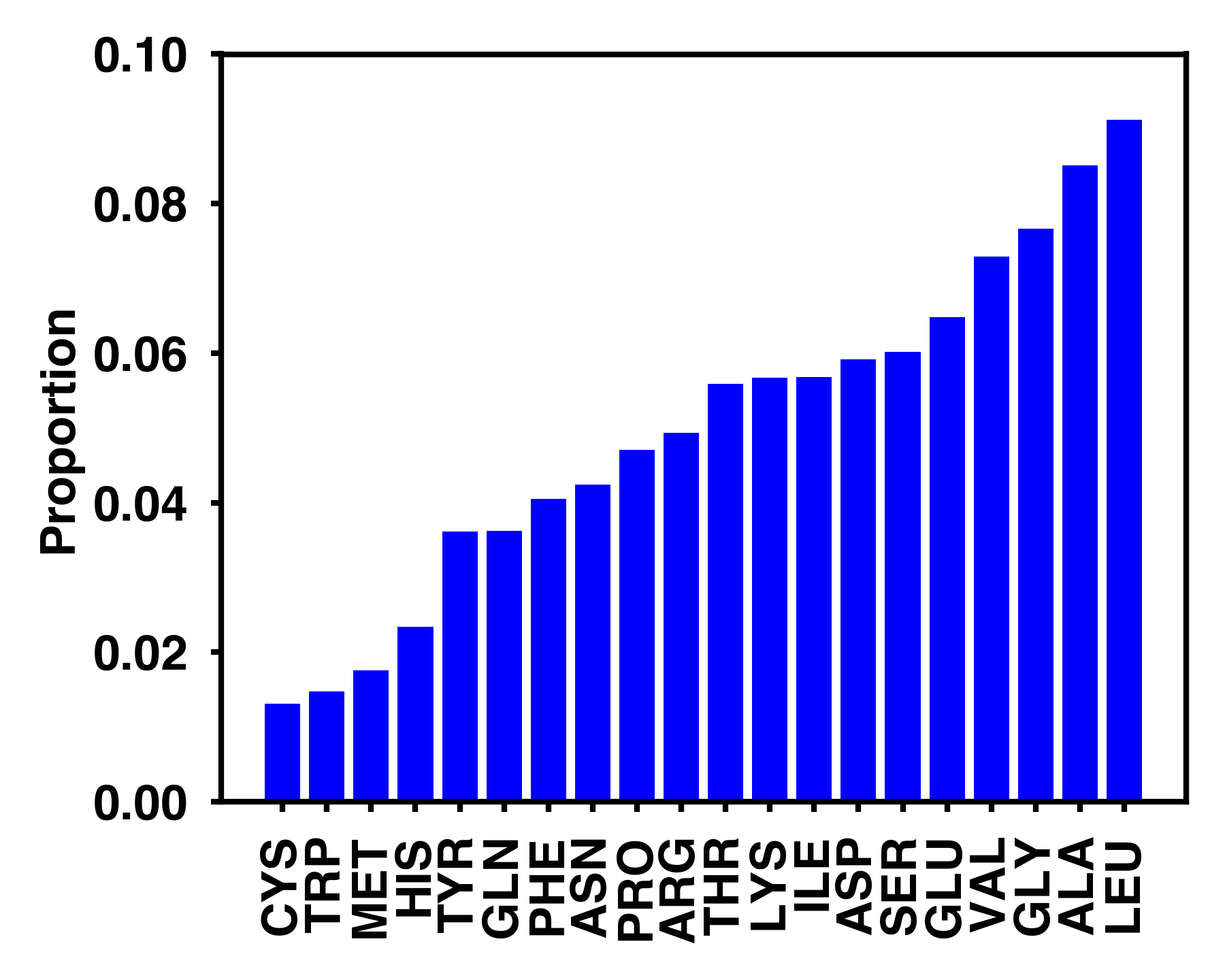


Figure S1. Amino acid composition in the SI90N15 training set.
